# Mesenchymal Stromal Cell-Based Therapies as Promising Treatments for Muscle Regeneration After Snakebite Envenoming

**DOI:** 10.3389/fimmu.2020.609961

**Published:** 2021-02-03

**Authors:** E. Eduardo Sanchez-Castro, Cecilia Pajuelo-Reyes, Rebeca Tejedo, Bárbara Soria-Juan, Rafael Tapia-Limonchi, Etelvina Andreu, Ana B. Hitos, Franz Martin, Gladys M. Cahuana, Clara Guerra-Duarte, Thamyres C. Silva de Assis, Francisco J. Bedoya, Bernat Soria, Carlos Chávez-Olórtegui, Juan R. Tejedo

**Affiliations:** ^1^ Faculty of Biological Sciences, Universidad Nacional Mayor de San Marcos, Lima, Peru; ^2^ Institute of Tropical Diseases, Universidad Nacional Toribio Rodríguez de Mendoza de Amazonas, Chachapoyas, Peru; ^3^ Faculty of Medicine, Universidad Privada San Juan Bautista, Lima, Peru; ^4^ Department of Molecular Biology and Biochemical Engineering, Universidad Pablo de Olavide, Seville, Spain; ^5^ Department of Surgery, Fundación Jiménez Díaz, Unidad de Terapias Avanzadas, Universidad Autónoma de Madrid, Madrid, Spain; ^6^ ISABIAL-Hospital General y Universitario de Alicante, Alicante, Spain; ^7^ Departmento de Fisica Aplicadas, University Miguel Hernández, Alicante, Spain; ^8^ Department of Cell Regeneration and Advanced Therapies, Andalusian Center of Molecular Biology and Regenerative Medicine-CABIMER, University of Pablo de Olavide-University of Sevilla-CSIC, Seville, Spain; ^9^ Biomedical Research Network for Diabetes and Related Metabolic Diseases—CIBERDEM, Instituto de Salud Carlos III, Madrid, Spain; ^10^ Center of Research and Development, Fundação Ezequiel Dias, Belo Horizonte, Brazil; ^11^ Departament of Biochemistry and Immunology, Institute of Biological Sciences, Universidade Federal de Minas Gerais, Belo Horizonte, Brazil; ^12^ Institute of Bioengineering, University Miguel Hernandez de Elche, Alicante, Spain

**Keywords:** advanced therapy medicinal products, mesenchymal stromal cells, snakebite, envenoming, muscle regeneration

## Abstract

Snakebite envenoming is a global neglected disease with an incidence of up to 2.7 million new cases every year. Although antivenoms are so-far the most effective treatment to reverse the acute systemic effects induced by snakebite envenoming, they have a limited therapeutic potential, being unable to completely neutralize the local venom effects. Local damage, such as dermonecrosis and myonecrosis, can lead to permanent sequelae with physical, social, and psychological implications. The strong inflammatory process induced by snake venoms is associated with poor tissue regeneration, in particular the lack of or reduced skeletal muscle regeneration. Mesenchymal stromal cells (MSCs)-based therapies have shown both anti-inflammatory and pro-regenerative properties. We postulate that using allogeneic MSCs or their cell-free products can induce skeletal muscle regeneration in snakebite victims, improving all the three steps of the skeletal muscle regeneration process, mainly by anti-inflammatory activity, paracrine effects, neovascularization induction, and inhibition of tissue damage, instrumental for microenvironment remodeling and regeneration. Since snakebite envenoming occurs mainly in areas with poor healthcare, we enlist the principles and potential of MSCs-based therapies and discuss regulatory issues, good manufacturing practices, transportation, storage, and related-procedures that could allow the administration of these therapies, looking forward to a safe and cost-effective treatment for a so far unsolved and neglected health problem.

## Introduction

Snakebite envenoming (SBE) is a public health problem affecting as many as 2.7 million people every year all around the world, most of whom live in poorly developed tropical and subtropical countries (1). Since 2017, SBE is considered a highest priority neglected tropical disease and the lack of enough antivenom supply in affected countries lead to thousands of deaths per year (2). Local damage, such as myonecrosis, dermonecrosis, hemorrhage, and edema, is only partially neutralized by antivenoms, at most. This is a noteworthy situation, considering that local damage can lead to permanent disability (3). Therefore, there is a current and urgent need of: i) providing adequate antivenom supply and ii) developing new comprehensive or complementary treatments that help to neutralize both systemic and local damage (and then permanent sequelae) (4).

Among the various tissue damage effects induced by SBE, myotoxicity-induced local myonecrosis (5) and hemorrhage (6) could be the most severe ones. Impaired skeletal muscle regeneration (SMR) (7, 8) may be associated or not to low response to antivenom (9).

SMR is a complex process with three overlapping steps (10), all of them affected by SBE. Mesenchymal stromal cells (MSCs) based therapies have shown to induce muscle regeneration in a variety of models in preclinical and clinical studies, mainly by anti-inflammatory activity, paracrine effects, revascularization induction, and microenvironment remodeling (11–14). In a recent publication, our group could demonstrate that adipose derived allogenic MSCs revert macrophage activation, cytokine storm and hyperinflammatory state associated with COVID-19 showing that MSCs stops tissue damage and promotes recovery (15). This promising landscape, considering the nature of the local and systemic damage induced by SBE, and the matching benefits of MSCs based therapies drive us to propose that this kind of treatment could be effective in reverting the induced tissue damage caused by SBE that do not respond to antivenom treatment, in particular by improving SMR.

Here, we will discuss the biological basis of local and systemic effects induced by SBE and the well-described benefits of MSCs based therapies in similar processes. Furthermore, preliminary results of MSCs’ secretome treatment upon muscle damage caused by *Bothrops atrox* venom are presented, supporting our approach. Also, we will discuss about regulatory issues, good manufacturing practices, transportation, storage, and related procedures that will be necessary to effectively translate this proposal into treatments for this neglected medical problem.

## Snakebite Envenoming (SBE) Relevance

SBE is a public health problem with at least 1.8–2.7 million of cases worldwide per year with mortality estimations ranging from 81,410 to 137,880 deaths (1). In the developing world, tropical and subtropical Africa, Asia, Oceania, and Latin America, SBE is considered a major public health problem with higher human morbidity and mortality ratios due to factors such as the scarcity of antivenom, poor health services, and problems in the transportation of patients (16). Furthermore, different types of sequelae are commonly presented after SBE, from psychological trauma to physical disabilities, with 400,000 of the surviving victims suffering from permanent sequelae (17–20). For instance, the annual number of amputations due to SBE in sub-Saharan Africa alone was estimated from 5,908 to 14,514 (21).

Currently, SBE is a Category A neglected tropical disease, according to the World Health Organization (WHO), which remarks the importance of SBE in terms of incidence and severity, especially for developing countries (2). Moreover, a Road Map for a globally coordinated response to SBE was recently published by WHO, with the ambitious goal of reducing snakebite deaths and disability by 50% before 2030 (4).

One of the main obstacles in providing adequate treatment for SBE is that venom composition is heterogeneous. As an example, in the two main venomous families (Viperidae and Elapidae), we may find a great diversity of toxin families, such as snake venom metalloproteinase (SVMP), phospholipase A2 (PLA2), snake venom serine proteinase (SVSP), three-finger toxin (3FTx), C-type lectin-like protein (CTL), L-amino acid oxidase (LAO), low molecular mass myotoxins (Myo), and others. This wide range of components contained in snake venoms; depending on the snake species, lead to complex local and systemic symptoms (22).

While the gold standard for treating SBE is based on specific antivenom, which have proved to be effective against systemic symptoms and can save the lives of affected people, SBE also leads to long-term sequelae and disabilities, many of them caused by local damage which is mainly neither prevented nor reversed by antivenoms (9). Despite the variety of toxins, their resulting local tissue damage usually includes myonecrosis, dermonecrosis, hemorrhage, and edema; and also can be accompanied by concomitant infection, compartment syndrome, and bite’s site complications (23). Moreover, local damage caused by SBE is frequently followed by poor tissue regeneration associated with tissue loss and dysfunction, leading to permanent sequelae as well as consequent social and psychological implication (8, 17, 20). It is noteworthy that SBE affects predominantly people at the economically productive age, increasing the economic impact of permanent disability (24). Even more, local damage could also lead to death when necrotizing fasciitis, a soft-tissue infection characterized by rapidly progressing inflammation and necrosis of subcutaneous fascial tissues, is developed (25), which makes the local damage and necrosis per se a life-threatening situation.

Local damage induced by SBE, particularly myonecrosis, is currently a worldwide unsolved and neglected medical problem. Recently proposed therapies, such as anti-inflammatory treatment for general local effects (26) or photobiomodulation (PBM) by low-level laser therapy for the sensory effects (27), are still insufficient, showing no significant advantage on SMR. However, PBM with light emitting diode (LED) in red and infrared wavelengths showed to be a more promising therapy, since it could reduce the extent of myotoxicity, edema, inflammatory infiltrate, and hyperalgesia in mice after SBE (28). Moreover, the accelerated tissue regeneration induced by PBM therapies has already been stated, and further studies are encouraged (29).

Another promising therapy regarding local damage is based on nanobodies (recombinant single-domain antigen-binding fragments from camelid heavy chain-only antibodies). Nanobodies are proposed to be used as antivenoms, showing notable advantages such as rapid diffusion, high-biodistribution due to their small size and no Fc region (responsible for adverse reactions in antivenom therapy) in their structure (30). A recent study demonstrated their efficacy in neutralizing both local tissue hemorrhage and myonecrosis in mice after SBE, when applied a mixture of selected specific nanobodies (31), showing the potential of this therapy over Fab or IgG- based antivenoms in resolving local damage.

In addition to antivenom, new therapies focused on local damage caused by SBE are required to prevent the consequent sequelae and disabilities. Since the effects caused by SBE are so heterogeneous, it is difficult to design a specific therapy for each particular toxin action. This is the reason why we propose that Advanced Therapy Medicinal Products (ATMPs) (comprising somatic cell therapies, gene therapies, and tissue-engineered products according to the EU Directive No 1394/2007) may be an alternative treatment for SBE, due to their properties. Specifically, we hypothesize that mesenchymal stromal cells (MSCs), activated by local signals from the venom-injured area, will display a broad range of therapeutic effects including anti-inflammatory, regenerative and disruptive of the complement-inflammation-coagulation crossroad, promoting not only tissue regeneration but also re-vascularization of the affected area. We also presume that cell-free MSCs-based therapies could also present these listed benefits, with the additional advantage of its production logistics being more adequate for use in low-income countries, which are the most affected by SBE.

## Local Myonecrosis Induced by Snakebite Envenoming

Local damage caused by SBE results in myonecrosis as one of their main complications. Although not yet completely understood, the current knowledge on the pathogenesis of snakebite-induced myonecrosis and its unsuccessful healing have been well described in a recent review (7).

Several snake venom components from the most medically relevant venomous snake families (Viperidae and Elapidae) contribute to myonecrosis, inducing both myotoxic and hemorrhagic damage. Muscle damage induced by SBE is a consequence of a direct action of myotoxins (such as PLA2, 3FTx, LAO, and Myo) upon the plasma membranes of muscle cells and an indirect effect in vascular degeneration and ischemia, in essence due to local hemorrhages caused by hemorrhagic toxins (such as SVMP, SVSP, and CTL) (32). Additionally, the unbalanced inflammatory response provoked by these toxins contributes to further tissue damage and impaired regeneration processes (33–35). All of these described deleterious effects are usually presented together in SBE victims, where myotoxins PLA2s and hemorrhagic toxins SVMPs are the primarily acting venom components (36).

PLA2s and PLA2 homologs, well-established mediators of myonecrosis (37, 38), interact with yet unidentified “acceptors” in skeletal muscle cell plasma membrane, causing rapid membrane disruption by both catalytically-dependent and independent mechanisms. For instance, a catalytically-active myotoxic PLA2 predominantly induces phosphatidylcholine hydrolysis, acting only on the external monolayer of the sarcolemma; whereas a catalytically-inactive PLA2 homolog does not depend on phospholipid hydrolysis to disrupt plasma membrane (39). Interestingly, cell membrane cholesterol content (inversely related to membrane fluidity) is a relevant proposed parameter for myotoxic effects due to PLA2s, considering that cell membrane cholesterol depletion (and increased membrane fluidity) promotes membrane damaging of myoblasts by this type of myotoxin (40). Finally, membrane perturbation caused by PLA2s induces a rapid influx of extracellular calcium which provokes hyper-contraction of myofilaments, mitochondrial calcium uptake (with mitochondrial damage due to calcium overload), and the activation of calcium-dependent intracellular proteinases (calpains) and even endogenous PLA2s themselves; defects that lead to myonecrosis (41).

SVMPs, zinc-dependent toxins can act directly at the microvascular level, causing hemorrhage (6, 42, 43). A unifying model explains their action mechanism (41). The hydrolyzation of basement membrane components of capillary vessels, especially type IV collagen, induces mechanical instability of the capillary (44, 45). Then, hemodynamic forces, such as wall hydrostatic pressure and shear stress, provoke the distention and disruption of the capillary wall, leading to extravasation (46). This damage indirectly contributes to myonecrosis by the restriction of oxygen and nutrients to muscle tissue (ischemia) (47). However, its most deleterious effect could come after myonecrosis, when hemorrhage hampers the regeneration process.

Although myotoxins such PLA2s are directly responsible for myonecrosis while hemorrhagic toxins such SVMPs indirectly contribute to this damage, it is remarkable how muscle regeneration in the presence of both types of toxins is significantly compromised, while in the presence of only PLA2, muscle recovers from myonecrosis without important abnormalities (8, 48). Considering that microvascularization is one of the determinant factors leading SMR (10), damaged microvasculature (specifically induced by hemorrhagic toxins) could be the main (but not the only) reason for poor muscle regeneration after SBE, in particular after Bothrops sp. (Viperidae snake) envenoming (38, 49, 50).

**Figure 1:**
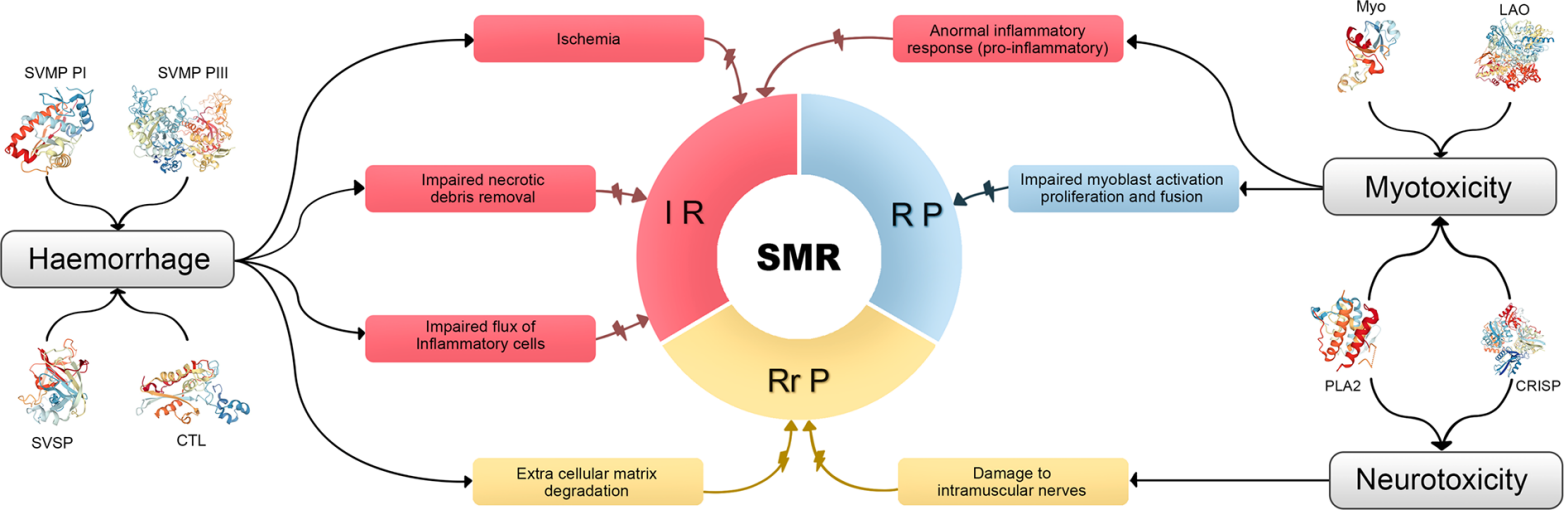
Summary of the main hypothetical factors that determine the poor outcome in skeletal muscle regeneration after myonecrosis induced by viperid venoms, associated to viperid toxins and the steps of the SMR which they affect. Different venom components induce hemorrhage, myotoxicity, or/and neurotoxicity. These deleterious effects impair the normal SMR acting on all their three steps. IR, inflammatory reaction; RP, regenerative/phase; RrP, remodeling-repair phase; CRISP, cysteine-rich secretory protein [Protein Data Bank accession ID (PDB ID): 3MZ8]; CTL, C-type lectin-like protein (PDB ID: 1IXX); LAO, l-amino acid oxidase (PDB ID: 2IID); Myo, low molecular mass myotoxin (PDB ID: 4GV5); PLA2, phospholipase A2 (PDB ID:1TGM for the monomer and PDB ID:3R0L for the dimer); SVMP, snake venom metalloproteinase (PDB ID:3DSL for class Plll and PDB ID: 1ND1 fo class PI); SVSP, snake venom serine proteinase (PDB 1D:1OP0).

However, other considerations beside myotoxic effects and microvascular damage must be taken into account when analyzing myonecrosis caused by SBE and its following regeneration. Damage of intramuscular nerves, degradation of muscle cell basement membrane, degradation of the extracellular matrix, and deleterious effects on myogenic cells are also involved (7). The **Figure 1**, summarizes the main hypothetical factors that determine the poor outcome in skeletal muscle regeneration after myonecrosis induced by viperid venoms, associated to viperid toxins and the steps of the SMR which they affect.

## Skeletal Muscle Regeneration (SMR)

**Figure 2:**
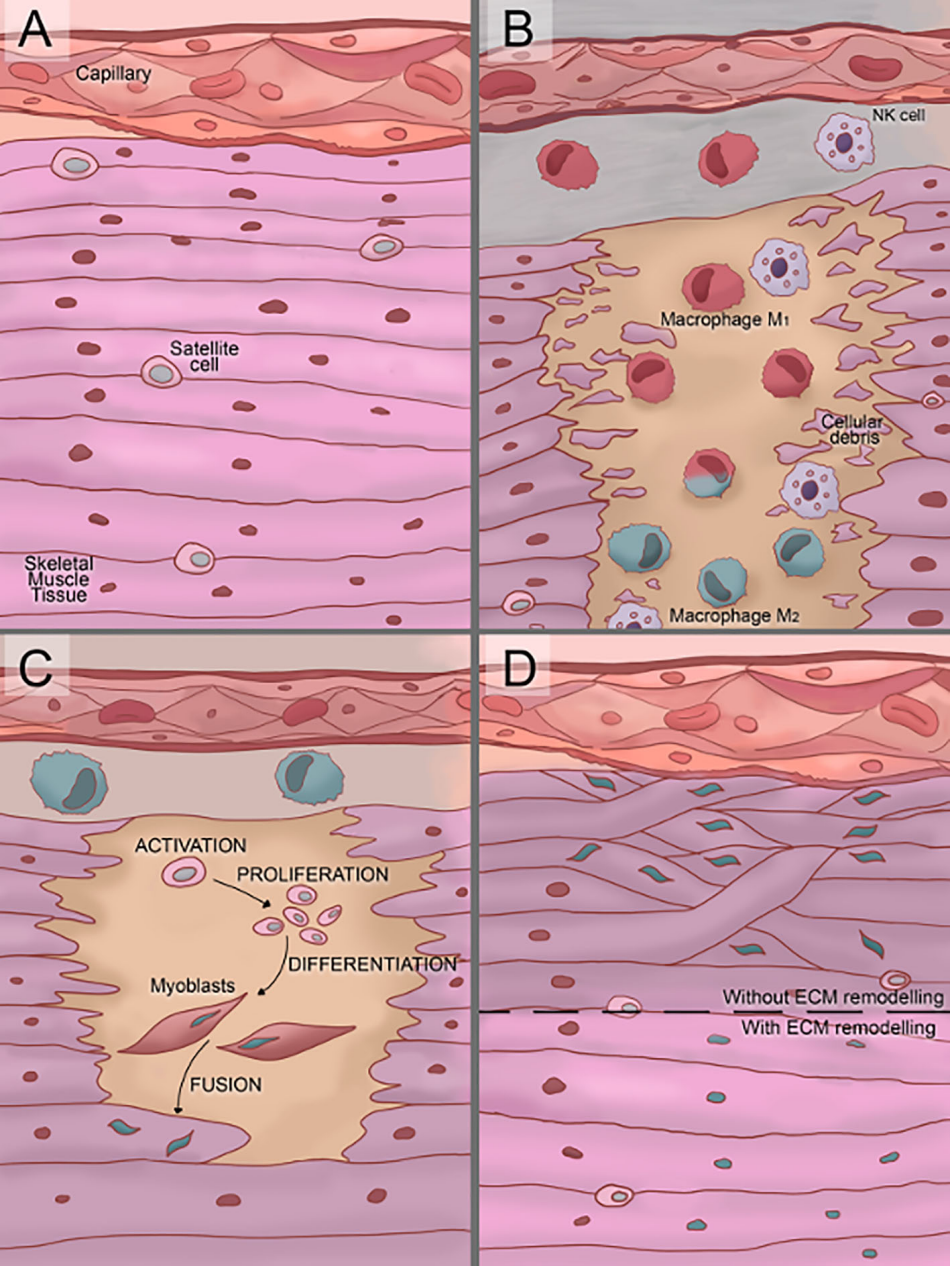
Illustration of the three phases of skeletal muscle tissue regeneration. **(A)** In normal conditions, satellite cells are located between the sarcolemma and basement membrane of terminally-differentiated muscle fibers. **(B)** Soon after damage, the inflammatory reaction starts. First, the cells of the immune system (neutrophils and macrophages) infiltrate the damaged tissue produce a proinflammatory stage. Over time, an anti-inflammatory stage starts to replace the proinflammatory one, in this transition, proinflammatory M1 macrophages switch to anti-inflammatory M2 macrophages. **(C)** Once the proinflammatory stage starts to decay, the regenerative phase starts with the satellite cells activation. Then, activated satellite cells proliferate and differentiate into myoblast and finally myoblasts fuse into myotubes. **(D)** The remodeling/repair phase consists in the extra-cellular microenvironment (ECM) remodeling and maturation of the new myofibers. If an inadequate remodeling/repair phase occurs, the myotubes grow unorganized generating dysfunctional muscle tissue.

SMR is defined as the multi-step process required for the formation of new myofibers or myofiber segments after necrosis. Three consecutives but overlapping stages are described (**Figure 2**) (10):

1. The inflammatory reaction, characterized by the infiltration of specialized cells (macrophages, neutrophils, etc.) which act as scavengers of necrotic debris and activate regulatory cells.
2. The regenerative phase, consisting in the activation, proliferation, differentiation, and fusion of satellite cells.
3. The remodeling-repair phase, including the maturation of newly formed myofibers and remodeling of regenerated muscle.

### Inflammatory Reaction

The inflammatory reaction is initiated by neutrophils in their early recruitment after myonecrosis and its induction is followed by macrophage activation (51). Even though phagocytes classical role was meant to be scavengers of necrotic debris, now it is known that they perform a more active role, orchestrating the inflammatory reaction and also promoting muscle regeneration (52, 53). The discovery of direct and constant heterocellular surface apposition over large areas and long linear distances between macrophages and myogenic cells throughout all stages of myogenesis reinforced their proposed importance not only at the inflammatory reaction but also at the regenerative phase (54).

A proper SMR depends on classical phagocytosis, which could allow the following stages of SMR after the inflammatory reaction; however, a precise balance between induced pro- and anti-inflammatory factors is also required (55). Here, macrophage subpopulations, M1 pro-inflammatory macrophages and M2 anti-inflammatory macrophages, play a pivotal role. While M1 macrophages release inflammatory mediators, including interleukin IL-1b, IL-12, and nitric oxide (NO) (56); M2 macrophages release anti-inflammatory mediators, including IL-4, IL-10, and transforming growth factor-beta (TGF-β) and promote remodeling of the extracellular matrix (ECM) and stimulate angiogenesis (57, 58). M2 macrophages also secrete insulin-like growth factor-1 (IGF-1) that activate muscle precursor cells (satellite cells) supporting their growth and fusion to form new muscle fibers (59). The benefit of using allogeneic MSC comes from the MSC-induced transdifferentiating of M1 (pro-inflammatory) into M2 (anti-inflammatory) macrophages.

Neutrophils are the first to reach the site of snake venom-induced tissue damage. They stimulate an inflammatory environment and after stopping myonecrosis, they participate in the regeneration of damaged tissue. The resolution of inflammation and tissue regeneration are mediated by the remotion of necrotic material and the release of chemokines, cytokines, and growth factors (60). In cancer, two different subtypes of neutrophil populations have been described (61). The N1 subtype is pro-inflammatory, mainly with phagocytic and cytotoxic activity. While, the N2 subtype is induced by TGF-β and a low level of IFN-β, reducing inflammation and releasing growth factors such as VEGF, promoting angiogenesis (61, 62). Is widely known that MSC produce TGF-β (63) and, recently, it was reported that MSCs stimulate the polarization to N2 subtype (64). Finally, MSC based therapies would favor the anti-inflammatory neutrophils subtype and tissue repair, in synergy with other regulatory cell populations also stimulated.

### The Regenerative Phase

The regenerative phase of SMR includes the activation, proliferation, differentiation, and fusion of satellite cells and ends with new functional myofibers. Satellite cells are the muscle precursor cells and they are required for a successful skeletal muscle regeneration (65, 66).

After muscle injury, satellite cells need to be activated and undergo a rapid proliferation for muscle regeneration. This activation is induced by different signals such as the generation of sphingosine-1-phosphate in the inner side of the plasma membrane of the satellite cell (67) or the increased NO synthase activity which generates more NO and probably induces the indirect release of hepatocyte growth factor (HGF) from the ECM (68, 69). In addition, several pro-myogenic stimuli that activate intrinsic pathways that stimulate proliferation are required, including IGF1, HGF, epidermal growth factor (EGF), fibroblast growth factor (FGF), tumor necrosis factor-α (TNFα) and β (TNFβ), platelet-derived growth factor-AA (PDGF-AA) and BB (PDGF-BB), vascular endothelial growth factor (VEGF) and also the implementation of a highly specialized, epigenetic and regulatory gene expression program (70–72). For instance, specific patterns of miRNAs (gene expression regulatory molecules) for regeneration and differentiation suggested they are likely involved in the process of satellite cell proliferation, differentiation, and skeletal muscle regeneration in general (73).

Activated satellite cells need to be differentiated into myoblast and consecutively form myotubes by fusion. For example, a required satellite cells activation factor such as HGF inhibits muscle cell differentiation (74). It was described that as well as the rapid proliferation stage is controlled by Notch signaling (75), Wnt signaling controls the differentiation phase (76). Different proteins such dysferlin, myomarker, Eps15 homology domain-containing proteins (EHD), and annexins have been associated with membrane myoblast fusion and myotube formation, undoubtedly the muscle cell communication through paracrine signaling, especially by exosomes (77), plays an important role for the correct satellite cell activation, differentiation, and maturation (78). Moreover, not only the protein cargo (such as VEGF or IGF1) is involved, but also miRNA cargo. More than 170 different miRNAs were found within muscle exosomes including miR-1, miR-133a, miR-133b, miR-206, which regulate myogenic differentiation/myoblast proliferation (79–82).

### The Remodeling-Repair Phase

Growth and maturation of newly formed myofibers may vary according to various factors from the type of damage to the involvement of blood vessels. However, it is remarkable that, after myogenesis, presence of the nerve is required. When neuromuscular connections are not reestablished, regenerating myofibers remain atrophic (83). Besides, another crucial factor for successful muscle regeneration is the maintenance of the basal lamina of muscle fibers, because remnants act as scaffolds to guide satellite cell divisions after injury (84). Also, the mechanical loading is essential for the subsequent maturation of myotendinous junctions and muscle remodeling; if immobilization is too prolonged, regenerated myofibers remain atrophic and their orientation is more disordered (10). Furthermore, mechanical loading enhances the ability of myoblasts to promote an M2-like macrophage phenotype following exposure to ECM scaffolds and M2-like macrophages promote myoblast chemotaxis and differentiation while lacking weight-bearing impairs muscle remodeling (85). This link between the inflammatory reaction and the remodeling-repair phase reaffirms muscle regeneration steps are overlapping and each one affects over the others.

In addition, a remarkable consideration for a proper SMR is the capillarization damage. Microvasculature would be a critical factor that transcends only one step of the SMR due to capillaries, satellite cells, and muscle remodeling appear to be intimately linked and capillarization would be necessary for appropriate necrotic debris scavenge, satellite cells function, systemic cytokines delivery, the transportation of muscle-derived cytokines, or for cell-cell interactions between satellite cells and endothelial cells (86).

However, even with all the current knowledge about the SMR, a well-described process, there is not much information about its occurrence after SBE. A successful SMR with proper inflammatory reaction, regenerative phase, and remodeling-repair phase require many factors such as the removal of necrotic material, the presence of intact blood supply and innervation, or the permanence of the basement membrane surrounding necrotic fibers (10). Unfortunately, after SBE, injured tissues do not provide the ideal conditions for SMR. Envenomed muscle tissue shows inhibited myoblast cell proliferation and fusion into myotubes (87). Moreover, the reported microvascularization and innervation damage after SBE may hamper a proper SMR (8). The implementation of a comprehensive treatment that could counteract the negative effects of SBE would be necessary to allow the normal SMR process and MSC-based therapies could accomplish these characteristics.

## Mesenchymal Stromal Cells-Based Therapies

MSC are multipotent stromal cells from different tissue sources that can be differentiated into a variety of cell types. The International Society for Cellular Therapy has proposed three minimum criteria to define MSCs (88): (i) adherence to plastic, (ii) specific surface antigen presence/absence, and (iii) expression of multipotent differentiation potential. Therefore, MSC-based therapies imply the direct or indirect use of MSC for therapeutic purposes. While cell-based therapies directly apply MSC to patients, cell-free therapies may use MSC conditioned medium containing the secretome (as “all the factors secreted by a cell”) or MSC derived exosomes (89), a part of this secretome that can be further isolated (90).

MSCs can be obtained from several sources (91). While source and purification protocol can modify MSC properties (92), in general, MSCs share several characteristics such as the fact that hypoxic culture enhances their proliferation (93) and that they have shown a well-studied therapeutic potential both in animals and humans (94–96).

**Figure 3:**
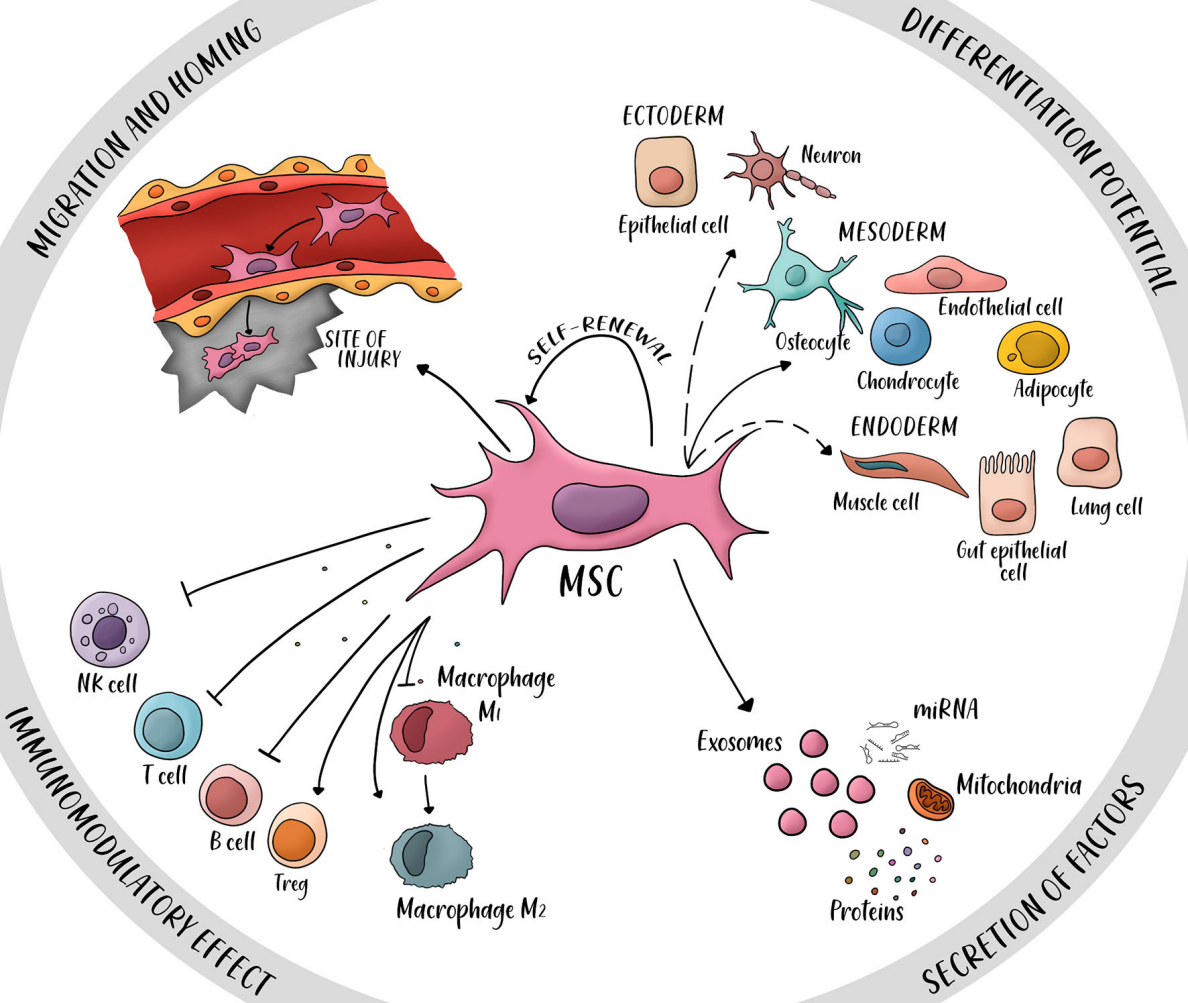
Four main properties of mesenchymal stem cells (MSC). MSCs have a potential for differentiation into ectoderm cells (neurons, epithelial cells), mesoderm (osteocyte, endothelial cell, chondrocyte, and adipocyte), and endoderm (muscle cells, gut epithelial cells and lung cells). Secretion of factors such as proteins, miRNAs, mitochondria, and exosomes can promote repair of damaged tissue and immunomodulatory potential. The immunomodulatory effect is mainly immunosuppressive, several secreted cytokines inhibit the activity of natural killer cells (NK cells), T cells and B cells; other cytokines activate the proliferation of regulatory T cells (T reg) and the switch from macrophage M1 (pro-inflammatory) to macrophage M2 (anti-inflammatory). The property of migration and homing is possible by the expression of specific ligands and receptors in the site of injury. Dashed arrows: Controversial transdifferentiation *in vivo*.

Four main properties have been proposed to explain MSCs therapeutic potential (**Figure 3**) (14): (i) the ability to secrete multiple bioactive molecules (proteins, mRNAs and miRNAs) capable of stimulating regeneration and inhibiting inflammation, (ii) the lack of immunogenicity and the ability to perform immunomodulatory functions, (iii) the ability to home to sites of inflammation following tissue injury when injected intravenously, and (iv) the ability to differentiate into various cell types.

**Figure 4:**
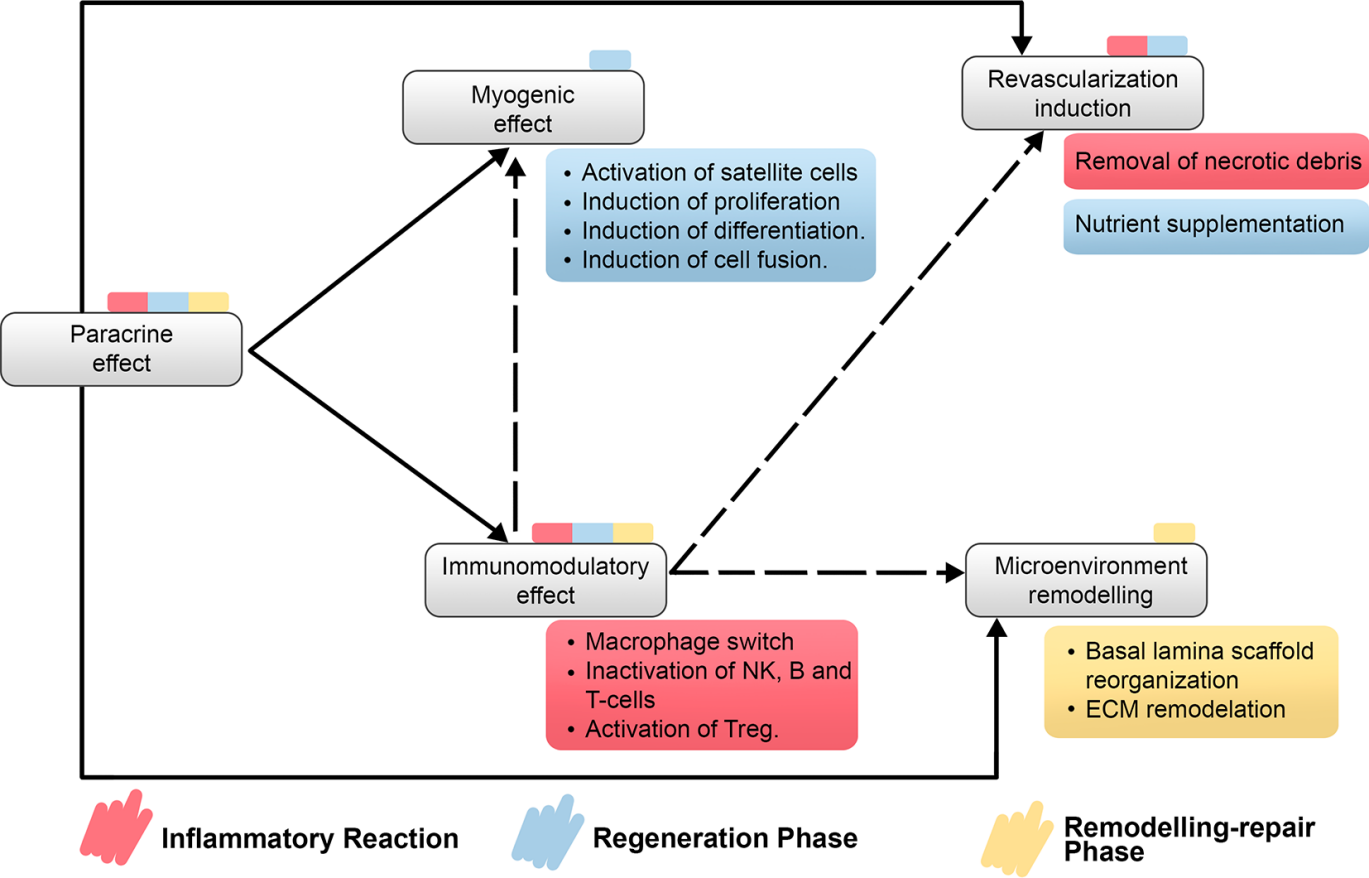
Relationship on main properties of MSC-based therapies regarding the recovery of the impaired SMR after myonecrosis induced by snakebite envenomation. It is detailed the direct and indirect relationship between properties of MSC-based therapies and which step or steps of SMR would be benefited by each property. Treg, regulatory T cells; NK cells, natural killer cells.

A relevant consideration when considering MSC-based therapies is their well-reported safety (97). Even though, some clinical trials have often shown only moderate success, which is usually attributed to low engraftment or low retention rates of cells as it is the case of most of MSC-based cardiovascular therapies, MSC-based therapies have been extensively reported to improve angiogenesis in both preclinical and clinical trials (13, 98–101). Moreover, reports of MSCs ability to induce tissue microenvironment remodeling directly over ECM components (12, 102) and MSCs immunomodulatory potential (103) reinforce the proposition that paracrine effects govern the therapeutic potential of MSC but also prompt the theory that MSC-based therapies could enhance SMR after SBE (**Figure 4**).

The therapeutically effect of MSC administrated by intrarterial or intravenous infusion or local injection has been demonstrated in humans for more than 400 reported Clinical Trials (ClinicalTrials.gov) including our group’s experience with more than 200 patients suffering multiple sclerosis (104), Chronically Limb Ischemia (99, 105–107), and COVID-19 (15). Moreover, a meta-analysis of randomized controlled trials, showed that MSC-cell based therapy increase ulcer healing, angiogenesis, and reduce amputation rate (100), although the mechanisms involved in tissue regeneration in the ulcers healing is an aspect that should be further studied.

**Figure 5:**
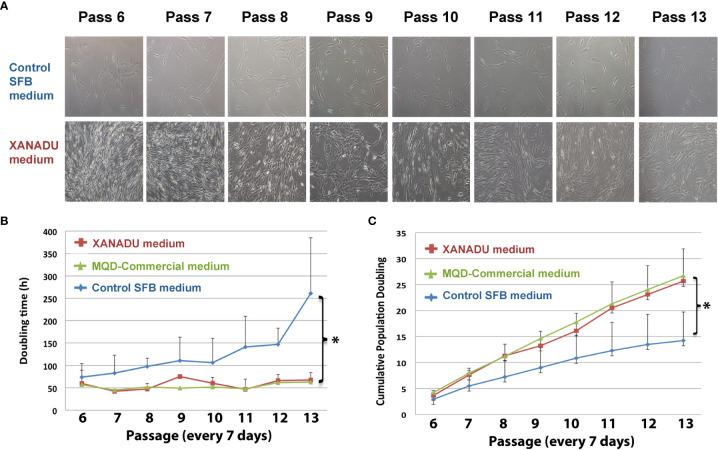
hMSCs derived from omentum adipose tissue, grown for long periods in chemically defined media have optimal morphological and proliferative properties. The hMSC were cultivated in control medium supplemented with fetal bovine serum (Control SFB medium) on adherent plates and XANADU chemically defined medium and commercial MQD on plates functionalized with vitronectin for eight consecutive passes. **(A)** Morphology and culture density of hMSC. **(B)** Duplication time. **(C)** Cumulative doubling of the population. The figures in **(A)** are representative of three independent experiments. The data of **(A, B)** are the mean plus the deviation of at least three independent experiments. (*) p ≤0.05 when compared to the control SFB medium.

Our group has designed a protocol for the isolation, characterization, and longtime culture of MSC derived from visceral fat using a xeno-free and human component free culture media (XANADU media, Patent pending). This protocol rejuvenates mitochondria in MSC with the advantages of i) not incorporating Fetal Bovine Serum (FBS) and avoiding the possibility of animal-to-human contamination and free of Human Platelet Lysate, which increases fibrinogen and prothrombosis. MSC obtained and expanded with this media express low Tissue factor and high tPA/PAI-1 ratio. Using XANADU media, adipose-derived human MSC expanded during eight consecutive passages (56 days, every 7 days), showing MSC characteristic morphology, without signs of replicative senescence (**Figure 5** panel A). The culture maintained a low doubling time (ranging from 45 to 70 h) similar to that obtained using a chemically defined commercial medium (MQD-commercial medium) and significantly less than those obtained with FBS-supplemented medium (FBS control medium) (**Figure 5**). The expansion of the adipose-derived human MSC in XANADU media shows a rate of proliferation suitable for its large-scale production (“biobanking”), due to the fact that the cumulative doubling of its population was significantly higher (25.6 h) than that obtained with control medium (14.21 h), (**Figure 5**) and similar to that obtained in commercial xeno-free media (**Figure 5**).

**Figure 6:**
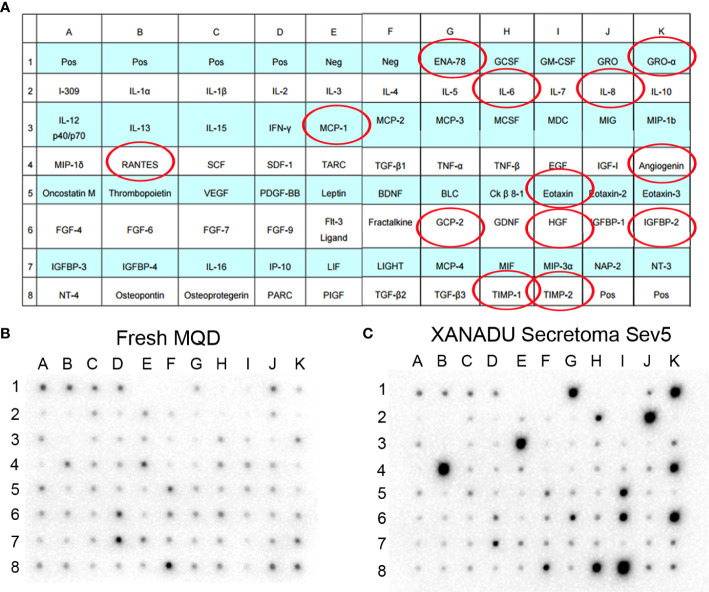
Secretoma of hMSC cultured in chemically defined medium. The hMSCs derived from omentum adipose tissue were isolated and cultured in XANADU chemically defined medium (hMSC- line Sev5). The obtained secretome was used for the assay of cytokine array. Both the fresh non-secretoma medium and the secretion-enriched medium of hMSC-line Sev5 were incubated on membranes of the Human Cytokine Antibody Array (Abcam) kit, following the manufacturers’ recommended protocol. The red circles indicate the cytokines that are significantly increased in the secretion-enriched XANADU after culture of the MSCs for 5 days compared to the fresh medium. The figure is representative of three independent experiments.

A preliminary study using a human cytokine antibody array shows that cells grown in XANADU media secrete paracrine factors with capacity for regulation of tissue regeneration and modulation of immune response, that may be relevant in the context of SBE (**Figure 6**). In particular, the enrichment of TIMP-1 and TIMP-2, natural inhibitors of matrix metalloproteinases (MMP) with an important role in setting the right balance for promoting tissue remodeling (7); Angiogenin, a powerful stimulator of angiogenesis; and RANTES, MCP1, ENA78, Gro-a, IL6, IL8, Eotaxin, GCP2 which are regulatory chemokine’s of the immune response.

Considering that SBE occurs primarily in developing countries, the benefits of treating the local damage with a cell-free MSC-based therapy are valuable. Therapeutic effects of MSCs are mostly mediated by their ability to produce bioactive molecules such as cytokines, growth factors, and extracellular matrix proteins, extracellular vesicles, and miRNAs. Since MSC-CM and MSC-exo carry the wide spectrum of secreted bioactive molecules, they are rational alternatives with several advantages compared to the direct use of MSC (108). MSC-EV has shown to induce accelerated SMR *in vitro* and *in vivo* by enhancing not only angiogenesis but also myogenesis (109). Therefore, cell-free MSC-based therapies appear to share the main properties of MSCs plus several advantages (110, 111). Some of them are (89, 112):

1. Reduced risks associated with engraftment.
2. Their lower immunogenicity compared with living cells.
3. Reduced possibilities of ectopic tissue development.
4. Significant lower cell number required for the treatments.
5. Their more cost-effective use of controlled laboratory condition (e.g., bioreactors) with easier and more productive procedures).
6. The possibility of being modified to desired cell-specific effects.
7. Their easier evaluation for safety, dosage, and potency.
8. Their convenient storage and transportation without altering their properties and without further precautions such as cryoprotectors.

Moreover, in the future, molecular engineering could modify the cargoes of MSC-S and MSC-EV to contain specific miRNAs, proteins, or surface markers to facilitate myogenesis and regeneration (78).

As stated above, bioactive molecules such proteins and miRNA are the effectors of the paracrine action produced by MSC (as well as MSC-S and MSC-EV). Some of these biomolecules can induce myogenesis by direct regulation of myogenic differentiation/myoblast proliferation, property that would enhance SMR acting at the regenerative phase (113). More important, antimicrobial peptides such as LL-37, hepcidin and β defensins inhibit microbial contamination in the injured tissue (therefore the life-threatening fasciitis) (114, 115). Some other molecules induce immunomodulation, revascularization, and microenvironment remodeling (14, 116–118), properties that would enhance SMR acting at all three steps of the process. These bioactive molecules appear to be signals with general effects on muscle cells, endothelial cells, and immune cells as we concern. Their effects improve the endogenous SMR in a comprehensive way which is ideal for treating local damage induced by SBE.

Immunomodulation is a property of MSC-based therapies. MSC express highly immunomodulatory markers such as CD73 (ecto-5- nucleosidase), IL-10, IDO, and other cytokines and interleukins, which inhibits the proliferation of T helper 2 and CTL lymphocytes, the effector function of inflammatory cells such as neutrophils and induce the proliferation of regulatory T cells (119–122). Also, this property induces the switch of macrophages from pro-inflammatory phenotype M1 to anti-inflammatory phenotype M2 (123), a key event during the inflammatory reaction of SMR. It is clear that during inflammation every type of regeneration is severely hampered, if it is even possible, so the macrophage switch is required for an efficient SMR; however, the role of M2 and M2-like macrophages transcend from the inflammatory reaction to the regenerative phase. They become activators of satellite cells by secreting bioactive molecules, which would mean that MSC-based therapies improve the regeneration by direct paracrine signaling but also by indirect paracrine signaling through macrophage secretions. This fact could imply a longer-lasting effect generated by MSC-S or MSC-EV even when these therapies do not maintain any living cellular medicaments in the damaged place, and then bypassing macrophage effectors. Moreover, immunomodulation is linked with the remodeling phase too by promoting remodeling of the ECM that returns to the regenerative phase of SMR because M2 macrophages need to interact with ECM to promote myoblast chemotaxis and differentiation. Also, angiogenesis is improved by M2 macrophages which made immunomodulation a property involved in all the three overlapping steps of SMR and also a particularly effective property against SBE. Immunomodulation helps to counteract SBE damage by preparing the damaged tissue with an appropriate anti-inflammatory microenvironment, activating precursor cells, recovering the ECM and inducing angiogenesis after microvasculature damage.

**Figure 7:**
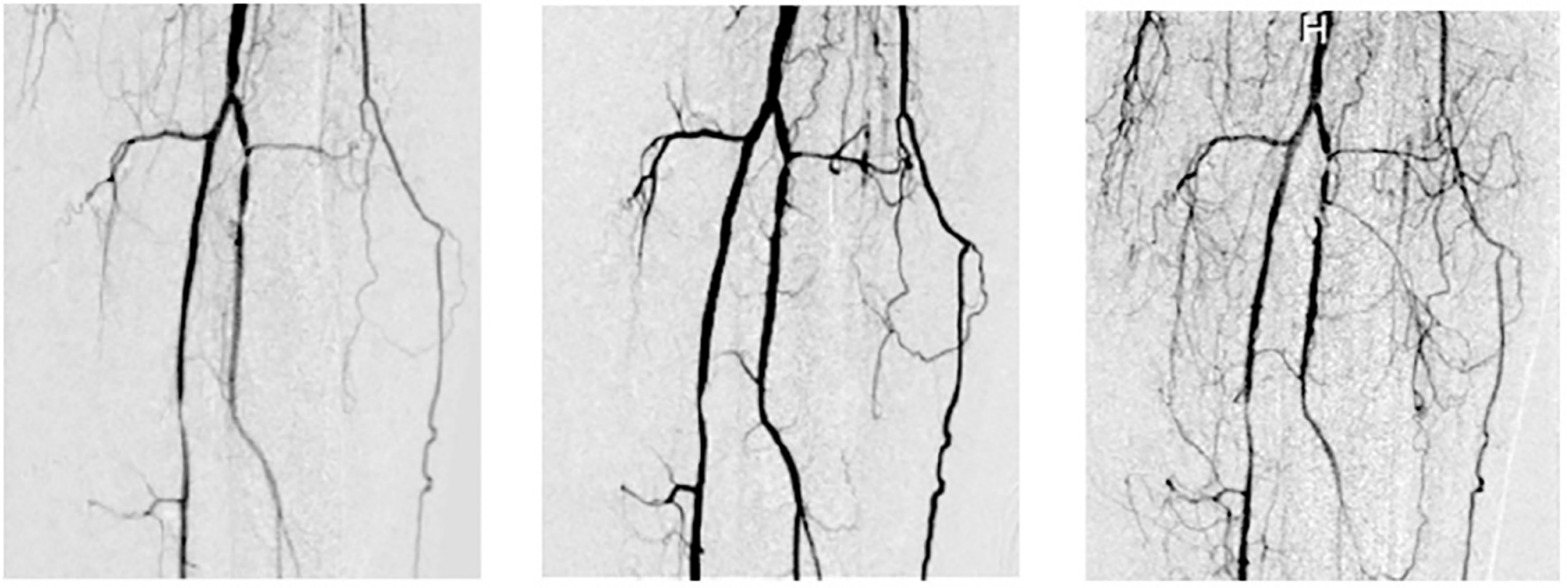
Vasculogenesis after Intrarterial adipose-derived MSC administration (intrarterial 1 x 106 cell/kg). Left: basal, middle: 6 months after and right: 12 months after intrarterial administration in a type 2 diabetic patient with a Grade 6 Rutherford (from Soria B. 2016. La Nueva Biología y sus Aplicaciones Médicas, with permission) (124).

Revascularization induction is another key property of MSC-based therapies that could help to improve SMR after SBE. Immunomodulation can help to induce angiogenesis and, it has been well-described as an effect of MSC-based therapies by our group (**Figure 7**). The presence of microvasculature in the place of local damage is necessary primarily due to its transportation implications. The inflammatory reaction implies the removal of necrotic debris and the regeneration phase needs a supply of nutrients during the activation, differentiation, and fusion of satellite cells. Also, the delivery of cytokines needs the presence of blood vessels as “highways”. Additionally, endothelial cells induce the regenerative phase by interacting with satellite cells. It is remarkable how the loss of microvasculature is a determinant reason why the SMR is impaired after SBE. Therefore, the positive effect of MSC-based therapies by enhancing angiogenesis and promoting neoangionesis could be one of the most important properties of these kinds of therapies.

Microenvironment remodeling induced by MSC-based therapies could be a decisive property for SMR after SBE. Considering that venom components completely disrupt the microenvironment of the damaged tissue, the remodeling phase may be impossible to perform without further stimulus. Although this step was enumerated as the last one of the three, the fact that SMR is an overlapping process means that it is necessary not only for a final result but also during the regenerative phase. Satellite cells need scaffolds to guide their divisions after injury and, even when newly myofibers are already differentiated and fused, their growth and maturation depends on ECM. By inducing microenvironment remodeling, MSC-based therapies cover a specially affected consideration after SBE and would improve the SMR till functional myotubes.

## Experimental Section: Assessing the Potential of MSC-Based Therapy in Envenoming Treatment

Aiming to further demonstrate that MSC’s derived products can have a therapeutical effect upon snake envenomings, we performed a pilot study using the secretome of adipose-derived human MSC cultured in XANADU culture media after intramuscular injection of *Bothrops atrox* venom in mice. This study was carried out in accordance with the principles of the Basel Declaration and recommendations of the Brazilian Council for the Control of Animal Experimentation (CONCEA). The protocol was approved by the Ethics Committee in Animal Use from the Federal University of Minas Gerais (protocol 321/2018-CEUA/UFMG).

**Figure 8:**
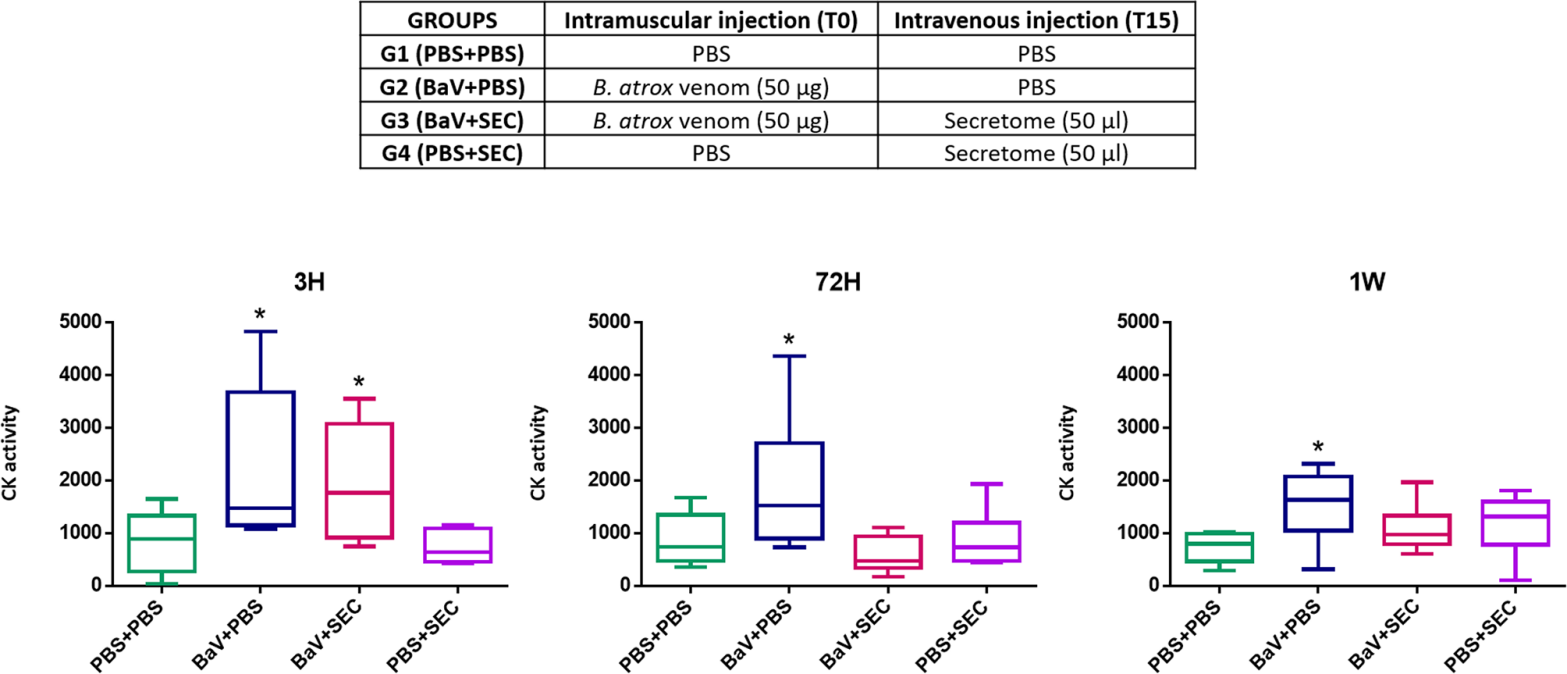
Preliminary assay. Creatine kinase levels of mice sera 3 h, 72 h, and 1 week after inoculation. Group PBS+PBS was injected with 50 µl of PBS by both intramuscular (i.m) and intravenous (i.v) routes. Group BaV+PBS received 50 µg of *Bothrops atrox* venom diluted in 50 µl of PBS and only PBS by i.v route. Group BaV+SEC was inoculated with 50 µg of *B. atrox* venom diluted in 50 µl of PBS i.m. and 50 µl of MSC secretome injected in the animal’s tail vein. Group PBS+SEC received PBS i.m and secretome i.v. * = p ≤0.05 when compared to the control PBS-PBS.

As stated above tissue damage, coagulopathies and hyperinflammation restricts the initiation of the regeneration process. **Figure 8** shows preliminary data on the acute effect of secretome administration on the muscle damage. Twenty 18–20 g female Swiss mice were divided in four groups of five animals each, according to the table below (**Figure 8**). As a negative control, a group of animals was injected with 50 µl of PBS by both intramuscular (i.m) and intravenous (i.v) routes (G1). To confirm the muscle damage caused by *Bothrops atrox* venom, a group received 50 µg of it diluted in 50 µl of PBS inoculated intramuscularly in the gastrocnemius muscle and, after 15 minutes, PBS by i.v route (G2). To simulate the treatment with MSC-based therapies we are suggesting in this review, a group of mice was inoculated with 50 µg of *B. atrox* venom diluted in 50 µl of PBS i.m. and, 15 minutes after the venom injection, 50 µl of the secretome was injected in the animal’s tail vein (G3). Finally, to assess whether the secretome alone had any effect by its own, another group received PBS i.m and secretome i.v (G4). Blood was collected from all animals by tail vein puncture 3 h, 72 h and one week after the injections. The sampled blood was tested for its creatine kinase (CK) activity, using CK-NAC kit, Labtest, Belo Horizonte. Brazil, as a biomarker of muscle injury. The test was made in duplicates and statistical analysis was performed using one-way ANOVA and Bonferroni post-test in GraphPad Prism software.

The results of this preliminary study (**Figure 8**) showed that animals from the groups inoculated with *B. atrox* venom had elevated CK levels three hours after venom inoculation, indicating muscle damage, as expected. This elevation was statistically significant (p ≤ 0.05) when compared to the control PBS-PBS. However, the group that received intravenous injection of secretome, 15 minutes after the venom injection, returned to CK levels comparable to controls 72 h after venom inoculation, whereas the group that received venom and intravenous PBS still presented higher levels at this time point. These results indicate a potential beneficial effect of secretome from MSC upon muscle damage caused by snake venom. They can be interpreted as an indication that the MSC secretome seems to reduce the extent of acute myonecrosis and that this may have an impact on reducing the extent of acute muscle damage and, probably, would favor a more successful regenerative response. The intravenous injection of secretome alone appears to have no effect on CK levels. These are still preliminary data that will be better and more deeply explored in the future but are an indication that the use of cell-free MSCs-based therapies have a promising potential as an alternative treatment for local damage caused by SBE. Pre-clinical and clinical trials of these therapies against snake venom are also mandatory before starting the translation process in a pilot study.

**Figure 9:**
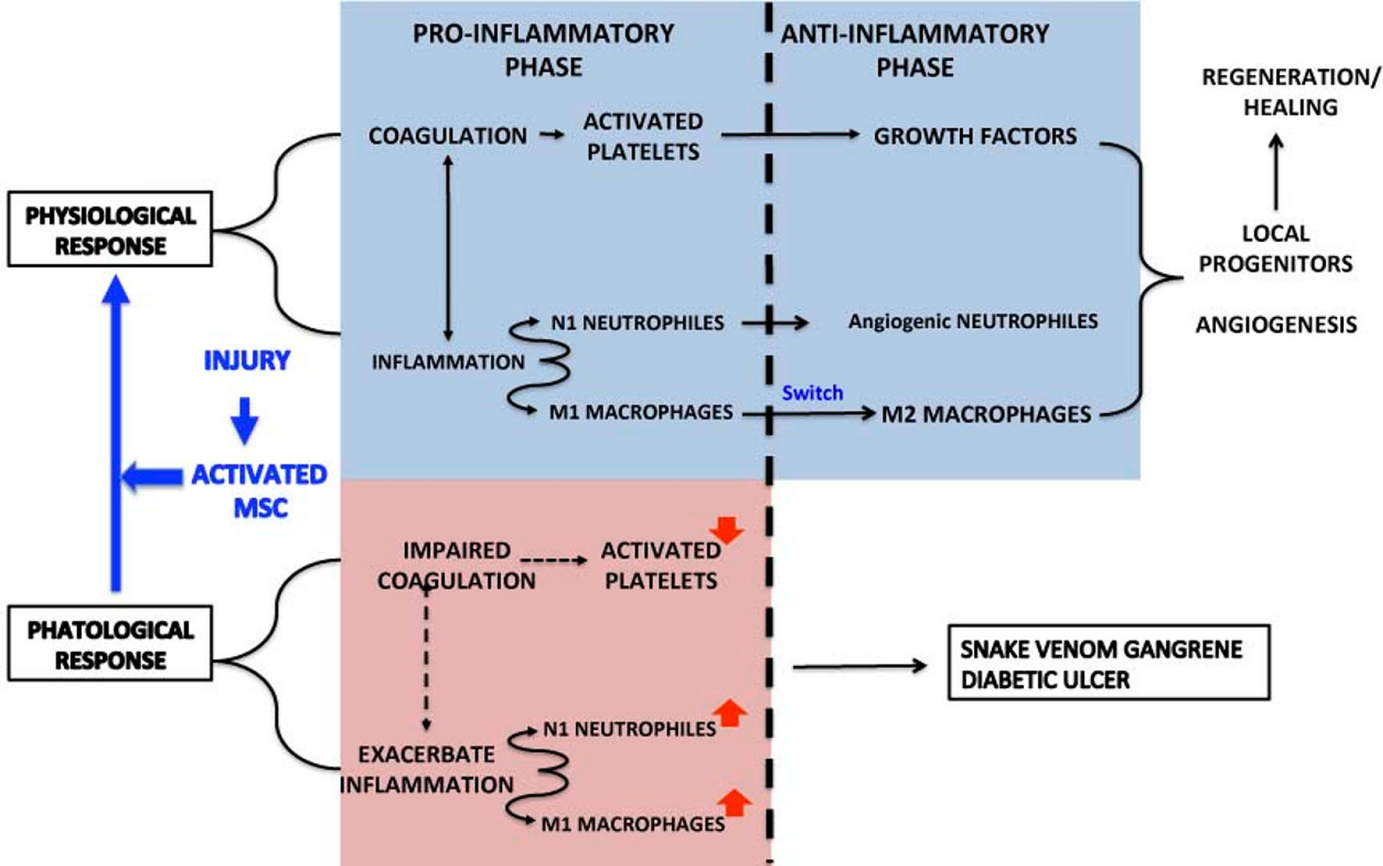
Summarizes the physiological and pathological response in the inflammation-regeneration crossroad. Under physiological circumstances the response to an injury activate platelets, N1 neutrophils and M1 macrophages. Platelets not only promote coagulation to stop hemorrhage but release growth factors that, in cooperation with Angiogenic neutrophils and M2 macrophages, contribute t tissue regeneration through the mobilization of local progenitors, angiogenesis and ECM remodeling.

In contrast, in certain processes such as Crohn’s complex fistulae, diabetic ulcers, and COVID-19, coagulation is impaired, inflammation exacerbated, M1 macrophages activated and releasing IL-1b, IL-12, and NO. The role of MSC (and here of the secretome in SBE) is to transform pathological inflammation into a physiological response (**Figure 9**). We anticipate that this approach will open a new avenue on tissue regeneration.

## The Time to Act Is Now: MSC-Based Therapies for Local Damage Induced by SBE

Advanced Therapy Medicinal Products (ATMP), including cell therapy, needs to meet some requirements to assure safety, efficacy, and quality in their use. While regulations specifics may vary among different locations, Good Manufacture Practices (GMP) are virtually a universal requirement for the production and application of ATMP such as MSCs based therapies (125–127). GMP manufactured cellular medicaments implies the definition of several variables such as cell dose and frequency, donor, cell source, culture process, isolation, and expansion. Related logistic such as storage (cryopreservation), transportation (cold chain), and quality tests are also necessary (96). For this specific case, considering that most SBE affected people reside in poor areas of developing countries, it is relevant to understand the viability of these alternatives.

As stated above, MSC-based therapies appear to be safe (97). Moreover, even when only moderate success or even failures could discourage their application, recent success and deeper comprehension of stem cell biology have provided a rationale pathway to MSC regulatory approval (128). Even for countries without any legislation about ATMP, as most developing countries, robust results based on pre-clinical and clinical trials following GMP would guarantee the application of these therapies. Therefore, the performance of pre-clinical and clinical trials is the key step for the future implementation of these alternatives.

It is a relevant question whether the application of these therapies is actually feasible. As GMP is not an extended practice in developing countries and the requirement of these ATMPs for the treatment of local damage induced by SBE implies the presence of the medication at remote places, it seems nearly impossible to perform them without an exorbitant local economic inversion. Luckily, the existence of cell-free therapies (MSC-S and MSC-EV) may improve this situation critically, by allowing the importation of ATMPs from foreign GMP compliant laboratories with relatively easy and cheap logistics. The absence of rejection problems allowing a status of universal compatibility of these therapies, together with their advantages at freeze-drying, packaging, and transportation (108) made these types of MSC-based therapies in promising prospects, even at precarious situations, because reduces costs and increase the applicability of these ATMPs not only to capital cities but to any level 2 hospital or superior.

Even when legal approval and extended application of MSC-based therapies for local damage caused by SBE is certainly years away from now, the characteristics and properties of MSC-based therapies, especially of cell-free therapies, for enhancing SMR while counteracting SBE effects would help millions of people who may avoid disabilities through the implementation of these treatments.

## Concluding remarks

In this article, we have proposed an alternative treatment for SBE, a highest priority neglected tropical disease affecting millions of people every year worldwide. Since the most affected people by SBE is part of the economically productive population suffering from bites during, ironically, their working time, giving them a therapy against a considerable risk of disability would impact both at public health and economic levels. Moreover, the WHO has reaffirmed the importance of this kind of proposals by announcing their goal of reducing snakebite disability by 50% before 2030.

While gold-standard treatments against SBE are antivenoms, which are effective against systemic symptoms, they can only neutralize partially local damage. MSC-based therapies could cover the lacking aspects related to SMR after SBE by counteracting the microvasculature and microenvironment damage as well as enhancing SMR after myonecrosis at every step of the regeneration process. Furthermore, MSC-S and MSC-EV could be excellent alternatives of cell-free therapies (with clear logistic advantages for the current case and the support of its preliminary successful results) since the positive effect of MSC-based therapies seems to depend on the secretion of bioactive molecules, property that both therapies share.

In summary, MSC-based therapies appear to be ideal, comprehensive alternatives for enhancing SMR after SBE due to their ability to act at all the three overlapping steps of SMR by paracrine effects, myogenic induction, immunomodulation, revascularization, and microenvironment remodeling, as well as they are likely to counteract the specific damages provoked by SBE. The development and optimization of MSC-based therapies for local damage induced by SBE could improve the quality of life of millions of people, especially people in developing countries, contributing to reduce public health burden and economic impact of this neglected tropical disease.

## Author Contributions

JT, CC-O, and BS conceived the concept of the paper. ES-C, CP-R, JT, CG-D, and BS wrote the first draft that was circulated, and all the authors contributed with different sections. CC-O, CG-D, and TCSA designed and executed the preliminary *in vivo* assay. AH prepared secretome samples and answered the second round of revisions. All the authors contributed to the acquisition, analysis, and interpretation of data for the work, revising it critically for important intellectual content, final approval of the version to be published, and agree to be accountable for all aspects of the work in ensuring that questions related to the accuracy or integrity of any part of the work are appropriately investigated and resolved. CC-O, CG-D, BS, and JT edited and submitted the final version of the manuscript. All authors contributed to the article and approved the submitted version.

## Funding

The authors are supported by the University Pablo de Olavide (Sevilla), the University Miguel Hernández (Elche, Alicante), National University Toribio Rodriguez de Mendoza (Chachapoyas, Peru) Grants: Contrato N° 09-2019-FONDECYT-BM-INC.INV to JRT, JDRF 2-SRA-2019-837-S-B and AVI-GVA COVID-19-68 to BS, Fundación Andaluza de I+D and Al-Andalus Biopharma Project (FAID-2018-1). The authors CC-O, CG-D, and TCSA were supported by Conselho Nacional de Desenvolvimento Científico e Tecnológico, Brazil (CNPq) (Process: 406163/2018-9), Coordenação de Aperfeiçoamento de Pessoal de Nível Superior, Brazil - CAPES (Program COFECUB Process: 88881.191812/2018-01) and by Fundação de Amparo à Pesquisa do Estado de Minas Gerais, Brazil (FAPEMIG).

## Conflict of Interest

The authors declare that the research was conducted in the absence of any commercial or financial relationships that could be construed as a potential conflict of interest.
